# Effects of pole dance on mental wellbeing and the sexual self-concept—a pilot randomized-controlled trial

**DOI:** 10.1186/s40359-023-01322-z

**Published:** 2023-09-14

**Authors:** Jalda Lena Pfeiffer, Setia Kati Sowitzki, Thomas Schäfer, Frank Euteneuer

**Affiliations:** Department of Psychology, MSB Medical School Berlin, Rüdesheimer Strasse 50, 14197 Berlin, Germany

**Keywords:** Pole dance, Sexual self-concept, Mental wellbeing, Body appreciation

## Abstract

**Background:**

Prior studies on beneficial effects of dance have primarily focused on traditional and expressionistic dance forms, neglecting newer dance styles like pole dance, which employ distinct techniques. The present pilot randomized controlled trial examined psychological and psychosexual effects of pole dancing.

**Methods:**

Fifty women were randomized to an eight-weeks pole dancing program or waitlist. The primary outcome was global mental wellbeing. Secondary outcomes included several dimensions of the sexual self-concept, as well as body appreciation and global self-esteem.

**Results:**

Compared to waitlist, the pole dance group showed an increase in mental wellbeing and improvements in sexual self-efficacy, sexual anxiety, sexual self-esteem, and body appreciation.

**Conclusions:**

Pole dancing may have broad psychological effects on both overall mental wellbeing and important domains of the sexual self-concept.

**Supplementary Information:**

The online version contains supplementary material available at 10.1186/s40359-023-01322-z.

## Background

Dance is one of the earliest ways of human expression. Being more than just a physical activity, dance includes sensations, emotions, and cognitions [1]. The variety of dances that have emerged over the past centuries all around the world allows for manifold influences on self-expression, motivation, and affects [2]. Yet, despite its positive value in all cultures and its positive effects on many physiological and psychological variables, dance has only recently been recognized as a form of therapy [3]. Dance was found to improve mental wellbeing and quality of life, enhance empathy and positive emotions as well as stress regulation and social competencies, and has positive effects on sexual health [1, 3–13]. Especially when dancing in groups, people reported a stronger feeling of relatedness to others, as they learned more about other people’s feelings and bodies when synchronizing the mutual movements [14]. The act of dancing is expected to be empowering [15]. It was suggested that the creativity of dancing and experiencing one’s body in a new way raises self-esteem and body image [11, 12].

While previous studies on dance therapy have focused mainly on traditional and expressionistic dances, newer forms of dance with ultimately different techniques have been widely neglected so far. One example of such new dances is pole dance (also referred to as pole fitness, pole sport, dirty (formerly exotic) pole dance, and pole [16]. While often being associated with exotic dance and sexually oriented activities, pole dance has become increasingly popular over the past decade, has paved its way into the recreational sector, and has let a variety of pole dancing classes emerge in fitness studio offers [17–19]. It combines power exercises with sensual movements, which is why it is suggested to have positive effects on several psychological outcomes [20]. One unique characteristic that distinguishes pole dance from other physical activities is the empowering and sexually liberating notion, as the increasing de-stigmatization of pole dancing challenges societal norms and constructions of female sexuality [17, 19, 21, 22]. Previous research suggested that pole dancing is not only associated with mental wellbeing, but also with variables of potential relevance for one`s sexual self-concept and sexual health respectively [18], thereby facilitating the embracement of sexuality [23].

To date, only a few studies have examined psychological and health-related effects of pole dance. In a qualitative study by Whitehead and Kurz [19], pole as a recreational activity was examined to define pole as either empowering or confirming of traditional gender roles and sexuality norms. The study showed that participants’ experiences of pole dancing as a form of art felt liberating and empowering in taking control over one’s body [19]. These findings were supported in a qualitative study by Nicholas et al. [20], who found that pole dance enables women to feel proud about their sexual side and to use this pride for developing both inner (mental) and outside (physical) strength. A pilot study by Nicholas [24], who asked beginners to do pole dance twice a week for eight weeks, revealed significant positive effects on self-perceptions, self-concepts, fitness, and body compositions. Because of the lack of quantitative research in this field, Nicholas [24] suggested to conduct randomized controlled trials (RCTs) to investigate the psychological and physical health benefits of pole more rigorously. Yet, although pole dancing has been shown to be related to mental wellbeing and several variables of potential relevance for the sexual self-concept (SSC), RCTs are still missing. The present pilot RCT aims to investigate psychological and psychosexual effects of pole dancing. The primary outcome was global mental wellbeing. Secondary outcomes were sexual self-efficacy and sexual anxiety, two major aspects of the SSC, as well as sexual motivation, sexual consciousness, body appreciation, and sexual self-esteem. In addition to sexual self-esteem, secondary outcomes also included global self-esteem to evaluate whether potential improvements in self-efficacy are domain specific. We hypothesized that eight weeks of pole dance training improves these variables. This trial may provide important implications for helping people to cope with both reduced mental-wellbeing and a poor sexual self-concept.

## Methods

### Participants

Participants were recruited via social media (e.g. Instagram and Jodel) and leaflets in fitness studios. Inclusion criteria were a minimum age of 18 years, female, native German speaker, and a prior professional experience of no more than two instructional pole dance sessions within the past six months or no more than four instructed lessons in total, respectively. To get trustworthy and externally valid results, we aimed for a minimum of 100 participants in each group. Similarly, a power analysis using GPower 3.1. [25] revealed a minimum sample size of *N* = 88 per group, when considering an effect of *d* = 0.5, Alpha = 5%, and 1-β = 0.95. Unfortunately, though we were not able to recruit this number of people, which was particularly due to the Corona-related restrictions during the time of data acquisition, so that we title and present our study as a pilot. The sample comprised 50 participants which were randomized to a pole dance group or a waitlist control group. Due to contact restrictions during the Corona pandemic, participants from the same households were paired up and considered as ‘one’ throughout the process of randomization. By doing so, participants were able to share a pole and assist each other, if necessary, without breaking Corona-related restrictions. Due to the nature of the study, no blinding of participants was possible. Prior to study participation, all participants provided written informed consent.

### Intervention

Pole dance sessions took place between October and December 2020 at two different pole dance studios in Berlin under real-life conditions. The intervention consisted of eight-week dance sessions given by a certified pole dance instructor with one session given once per week. A session took 60 min in total and was divided into 30 min of strength, endurance, and flexibility exercises, and 30 min of pole dance specific training. The latter consisted of spinning (“spins”) and static figures, as well as floor elements (see supplement A). The elements in the program did not vary inter-individually. However, some adjustments were made to match an individual’s abilities; hence regressions (i.e., a slightly easier variation of a figure) and progressions (i.e., a slightly more difficult variation of a figure) of the planned elements were implemented to ensure an adequate learning environment. Due to COVID-19 pandemic restrictions, the originally planned group classes became unfeasible. As a result, participants received personal training on the pole, either individually or per household. In addition, they received weekly videos with exercises for strength, endurance, and flexibility training, as well as “Sensual Pole” exercises. Data collection took place before randomization (baseline) and at the end of the intervention (post-intervention). As initially advocated, participants of the control group were given the choice of joining pole dance classes after the end of the intervention. The participation in pole dance classes was for free. However, all individuals who participated in pole dance sessions contributed an amount of 12 Euro to the room rental.

### Assessment

#### Mental wellbeing

Wellbeing was assessed using the German Version [26] of the Warwick Edinburgh Mental Wellbeing Scale (WEMWS) [27]. The WEMWS consists of 14 items with a five-point Likert response format ranging from (1) “none of the time” to (5) “all of the time”. Higher scores indicate higher mental wellbeing. Both content validity and test-retest reliability are high [28–30].

#### Multiple aspects of the sexual self-concept (SSC)

The Multidimensional Sexual Self-Concept Questionnaire (MSSCQ) was used to measure multiple aspects of SSC [31]. SSC refers to a person’s understanding of their sexual feelings, cognitions, and behaviors [31]. Every person is presumed to have a mental image of the sexual self in mind [32]. The MSSCQ has been validated in several populations [31, 33–35]. For the present purpose, we used the MSSCQ subscales for sexual self-efficacy and sexual anxiety, as well as subscales for sexual motivation, sexual consciousness, body appreciation, and sexual self-esteem. Sexual anxiety involves the tendency to feel anxious and uncomfortable in sexual situations. Sexual self-efficacy comprises the individual beliefs about controlling and realizing behaviors and affective responses within a sexual context in a successful manner. Sexual self-esteem encompasses the positive evaluation of the sexual self which enables the experience of a healthy and satisfying sexual life. Sexual motivation describes the urge to engage in sexual behavior while sexual consciousness is described as the attention to internal private bodily sensations associated with sexual arousal and motivation [31, 36]. Using a five-point Likert response format, participants are required to indicate how strongly the agree or disagree with a statement [31]. Each subscale consists of 5 items with scores ranging from (0) “not at all characteristic of me” to (4) “very characteristic of me”. Subscale scores are calculated by averaging the items of each subscale.

#### Self-esteem

Rosenberg’s self-esteem scale [37] was used to assess global self-esteem. The questionnaire consists of 10 items with a four-point Likert response format ranging from (1) strongly disagree to (4) strongly agree. The German version of the test has high reliability and adequate convergent validity [38, 39].

#### Body appreciation

Body appreciation was assessed using the German version of the Body Appreciation Scale (BAS) – 2 [40]. The scale consists of 13 items assessing acceptance, respect and attention given towards one’s body and its needs. The BAS-2 uses a five-point Likert response format ranging from (1) never to (5) always. Reliability has been found to be very good and there is evidence for moderate construct validity [41, 42].

### Data Analysis

Baseline measures are reported as means with standard deviations. Intervention effects were assessed by estimating mean between-group differences in change scores (unstandardized estimates, B) with corresponding bootstrapped 95% confidence intervals and one-tailed p-values. Unstandardized estimates were converted to Cohen’s *d* as effect size [43]. All analyses were conducted on an intention-to-treat basis using maximum likelihood estimation. Statistical analyses were carried out with Mplus7 (Muthén & Muthén, 1998–2012).

## Results

**Fig. 1:**
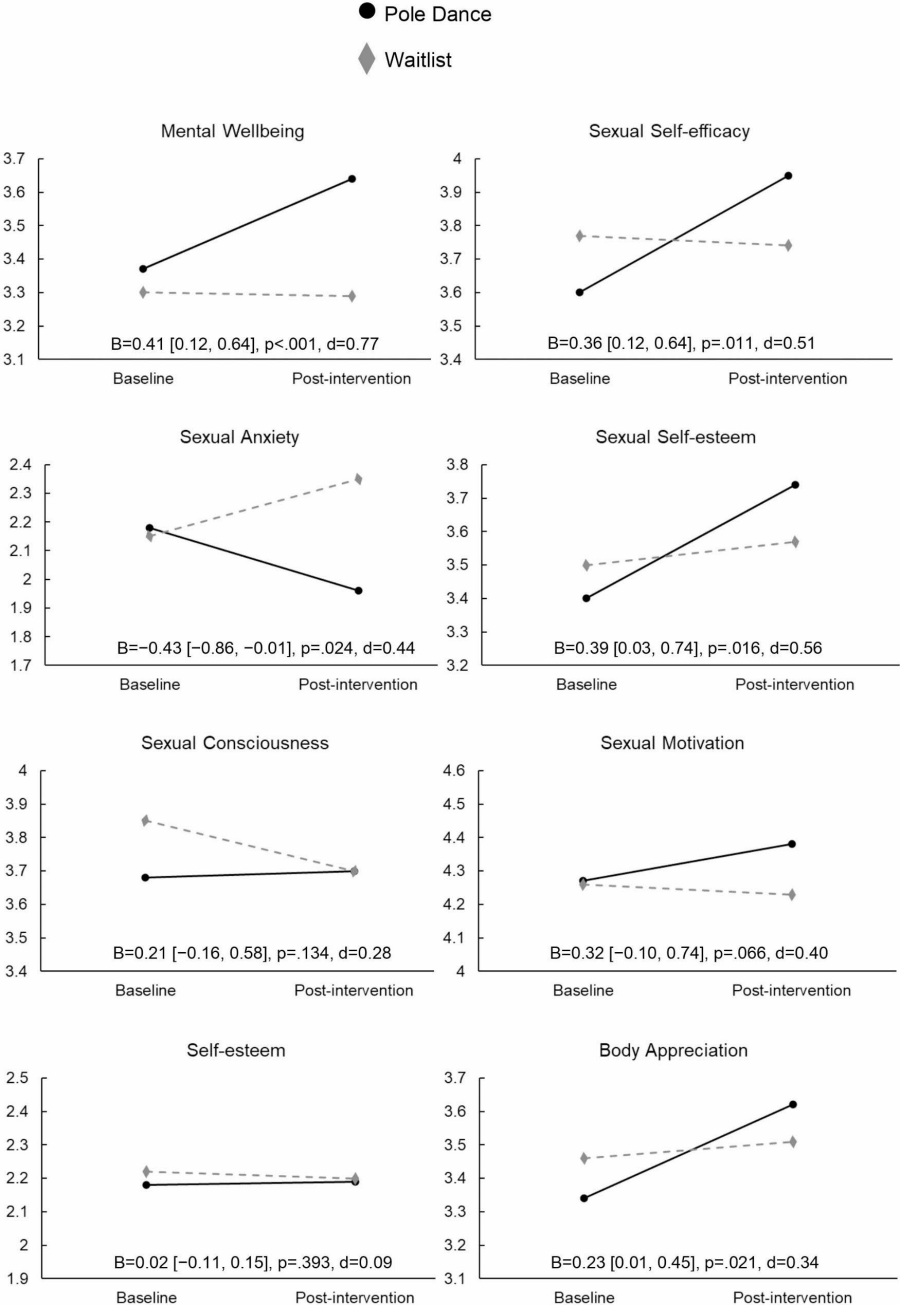
Changes from baseline to post-intervention for both treatment groups. Intervention effects are expressed by the unstandardized estimated mean differences in changes of outcome scores (B) with corresponding 95% confidence intervals in brackets, one-tailed p-values, and effect sizes (d)

Table 1 illustrates descriptive statistics for the two groups. Dropout rates from baseline to the end of the intervention were 15.4% for the dance group and 12.5% for the waitlist group (see supplement B for study flow). Figure 1 shows all changes from baseline to post-intervention and corresponding test statistics for between-group differences in change scores. Compared to the waitlist group, mental wellbeing increased in the pole dance group. In terms of secondary outcomes, the poledance group showed improvements in sexual self-efficacy, sexual anxiety, sexual self-esteem, and body appreciation, compared to the waitlist group. No intervention effects were observed for global self-esteem, sexual motivation, and sexual consciousness.

**Table 1:** Descriptive Statistics (M and SD)

Variable	Pole dance (*n* = 26)	Waitlist (*n* = 24)
Age	23.9 (2.75)	23.2 (2.69)
Mental Wellbeing		
Baseline Post-intervention	3.37 (0.54) 3.64 (0.42)	3.30 (0.56) 3.29 (0.63)
Sexual Self-efficacy		
Baseline Post-intervention	3.60 (0.61) 3.95 (0.67)	3.77 (0.71) 3.74 (0.73)
Sexual Anxiety		
Baseline Post-intervention	2.18 (0.95) 1.96 (0.81)	2.15 (1.07) 2.35 (1.13)
Sexual Self-esteem		
Baseline Post-intervention	3.40 (0.89) 3.74 (0.71)	3.50 (0.73) 3.57 (0.68)
Sexual Consciousness		
Baseline Post-intervention	3.68 (0.59) 3,70 (0.86)	3.85 (0.61) 3.70 (0.64)
Sexual Motivation		
Baseline Post-intervention	4.27 (0.92) 4.38 (0.76)	4.26 (0.85) 4.23 (0.83)
Self-esteem		
Baseline Post-intervention	2.18 (0.24) 2.19 (0.23)	2.22 (0.25) 2.20 (0.22)
Body Appreciation		
Baseline Post-intervention	3.34 (0.67) 3.62 (0.62)	3.46 (0.74) 3.51 (0,73)

## Discussion

The present randomized controlled trial examined psychological effects of pole dance. Despite its increasing popularity [20], pole dancing as a form of physical activity still lacks longitudinal and especially quantitative randomized controlled studies. In the present trial, several psychological effects were observed in the pole dance group compared to a waitlist control condition. These effects include improvements in general mental wellbeing, sexual self-efficacy, and body appreciation, as well as a reduction in sexual anxiety. In terms of self-esteem, the effect of pole dance was specific for sexual self-esteem, while changes in global self-esteem were not significantly different between both groups. No treatment effects were found for sexual motivation and sexual consciousness. These results indicate that pole dance may have broad psychological effects on both overall mental wellbeing and important domains of the SSC.

In the present study, the largest effect was observed for the primary outcome of mental wellbeing. These results support previous findings from qualitative research suggesting that pole dance may be empowering for women [19]. Although our study did not focus on specific mechanisms which may underlie an improvement in mental wellbeing, several factors may contribute to this effect. As pole dance trains strength, flexibility, and conditioning [16], it may lead to greater body control and respect for one’s body as well as improved self-confidence [20]. In addition, several psychological and biological mechanisms which have been suggested for mental health effects of exercise in general [44–46], may also be of relevance in the context of pole dance. Future studies should thus examine potential mechanisms of change with a focus on pathways, which may be more specific for pole dancing.

Pole dance has become a popular and accessible amateur physical activity and is characterized by its sexually liberating notion and increasing societal de-stigmatization [17, 19, 21, 22]. Due to the unique sexual properties of this particular dance style [17–19], we hypothesized an impact on the SSC that could potentially entail therapeutic implications for cases in which a modification of relevant dimensions of the SCC could be beneficial. While positive effects on mental wellbeing have been documented for several mind-body and exercise interventions [47, 48], our results may thus suggest that pole dance may help people to cope with sexual anxiety or a lack of sexual self-efficacy and self-esteem respectively. Importantly, both dance and sexual behaviors use the body to initiate and create movements [49]. Dance has early been reported to be a viable tool in psychological body-centered sexuality therapy as the body and mind were assumed to be intertwined and inseparable [4, 8]. Through the implementation of experiential and sensory elements, dance interventions were found relevant in facilitating a healthy sexuality [4, 8, 50]. Therefore, it seems likely that pole dance has the potential to facilitate a more positive SCC which precedes sexual behaviors and attitudes [31, 51]. Future trials with active control conditions and multi-centre samples are necessary to strengthen these assumptions.

Notwithstanding the strengths of the present study, such as the randomized controlled.

design and its novelty with respect to the area of pole dance, limitations need to be reflected. First, the regulations during the COVID-19 lockdown did not allow for group classes. Therefore, a putative socialization effect mentioned in previous studies [20] might have been reduced. Second, we did not assess participants’ compliance with respect to the additional videos for training at home. It is possible that not every participant completed the workouts, which might have led to fewer improvements during the classes and hence throughout the course of the intervention. Third, we did not assess long-term effects since no follow-up assessments were conducted. Finally, we did not include an active control condition, which limits conclusions about specific effects of pole dancing.

## Conclusions

To conclude, our study provides evidence that pole dancing may have broad psychological effects on both overall mental wellbeing and important domains of the sexual self-concept. Pole dancing may be useful to help people cope with sexual anxiety or a lack of sexual self-efficacy and self-esteem.

### Electronic supplementary material

Below is the link to the electronic supplementary material.

Supplementary Material 1

Supplementary Material 2

## Data Availability

Data are available on request from the corresponding author.

## References

[1] Adam D, Ramli A, Shahar S, Koch SC, Aguiar LPC, Da Rocha PA (2016). The mind–body connection in Dance/Movement Therapy: theory and empirical support. Am J Danc Ther.

[2] Sturm I, Baak J, Storek B, Traore A, Thuss-Patience P (2014). Effect of dance on cancer-related fatigue and quality of life. Support Care Cancer.

[3] Strassel JK, Cherkin DC, Steuten L, Sherman KJ, Vrijhoef HJM (2011). A systematic review of the evidence for the effectiveness of dance therapy. Altern Ther Health Med.

[4] Kierr S (2011). Is Dance/Movement Therapy relevant to the process of achieving a healthy sexuality?. Am J Danc Ther.

[5] Trautmann-Voigt S, Tanztherapie (2003). Zum aktuellen diskussionsstand in Deutschland. Psychotherapeut.

[6] Barton EJ (2011). Movement and Mindfulness: a formative evaluation of a Dance/Movement and yoga therapy program with participants experiencing severe Mental illness. Am J Danc Ther.

[7] Koch SC, Riege RFF, Tisborn K, Biondo J, Martin L, Beelmann A. Effects of Dance Movement Therapy and Dance on Health-Related psychological outcomes. A Meta-analysis update. Front Psychol. 2019;10. 10.3389/fpsyg.2019.01806 PMC6710484 31481910

[8] Reitz G (1998). Human-structural dance therapy and the development of body-ego-identity and sexuality. Dyn Psychiatry.

[9] Savidaki M, Demirtoka S, Rodríguez-Jiménez RM. Re-inhabiting one’s body: a pilot study on the effects of dance movement therapy on body image and alexithymia in eating disorders. J Eat Disord. 2020;8. 10.1186/s40337-020-00296-2 PMC7212562 32426135

[10] Takahashi H, Matsushima K, Kato T (2019). The effectiveness of Dance/Movement Therapy Interventions for Autism Spectrum disorder: a systematic review. Am J Danc Ther.

[11] Kiepe MS, Stöckigt B, Keil T (2012). Effects of dance therapy and ballroom dances on physical and mental illnesses: a systematic review. Arts Psychother.

[12] Muller-Pinget S, Carrard I, Ybarra J, Golay A (2012). Dance therapy improves self-body image among obese patients. Patient Educ Couns.

[13] Cruz RF (2018). Marian Chace Foundation lecture: rhythms of Research and Dance/Movement Therapy. Am J Danc Ther.

[14] Purser A (2017). Dancing intercorporeality: a Health Humanities Perspective on Dance as a Healing Art. J Med Humanit.

[15] Valentine GE. Dance/Movement Therapy with Women Survivors of Sexual Abuse. In: Brooke S, editor. The Use of Creative Therapies with Sexual Abuse Survivors. 2007.

[16] Holland S. Pole Dancing, empowerment and embodiment. Palgrave Macmillan; 2010.

[17] Kim Y, Kwon SY. I’m a poler, and proud of it: south korean women’s managed experiences in a stigmatized serious leisure activity. Soc Sci. 2019;8.

[18] Ołpińska-Lischka M, Kujawa K, Laudańska-Krzemińska I, Maciaszek J (2020). Pole Dance - A Way to boost the sense of sexual attractiveness or body Acceptance. Acta Kinesiol.

[19] Whitehead K, Kurz T (2009). Empowerment and the pole: a discursive investigation of the reinvention of pole dancing as a recreational activity. Fem Psychol.

[20] Nicholas JC, Dimmock JA, Donnelly CJ, Alderson JA, Jackson B (2018). It’s our little secret … an in-group, where everyone’s in: females’ motives for participation in a stigmatized form of physical activity. Psychol Sport Exerc.

[21] Choi D, DeLong M (2019). Defining female self sexualization for the Twenty-First Century. Sex Cult.

[22] Just SN, Muhr SL (2020). Holding on to both ends of a pole: empowering feminine sexuality and reclaiming feminist emancipation. Gend Work Organ.

[23] Pellizzer M, Tiggemann M, Clark L (2016). Enjoyment of Sexualisation and positive body image in recreational Pole Dancers and University students. Sex Roles.

[24] Nicholas J. The psychological, physiological, and injury-related characteristics of recreational pole dancing. University of Western Australia; 2019.

[25] Faul F, Erdfelder E, Lang A-G, Buchner A (2007). G*Power 3: a flexible statistical power analysis program for the social, behavioral, and biomedical sciences. Behav Res Methods.

[26] Bachinger A, Lang G. Warwick-Edinburgh Fragebogen zum psychischen Wohlbefinden. Forschungsinstitut des Roten Kreuzes. 2013.

[27] Stewart-Brown S, Janmohamed K. Warwick-Edinburgh Mental Well-being scale (WEMWBS) - user guide. Warwick; 2008.

[28] Lang G, Bachinger A (2017). Validation of the german Warwick-Edinburgh Mental Well-Being scale (WEMWBS) in a community-based sample of adults in Austria: a bi-factor modelling approach. J Public Health (Bangkok).

[29] Stewart-Brown S, Janmohamed K. Warwick-Edinburgh Mental Well-being Scale (WEMWBS) - User Guide. 2008.

[30] Tennant R, Hiller L, Fishwick R, Platt S, Joseph S, Weich S et al. The Warwick-Dinburgh mental well-being scale (WEMWBS): development and UK validation. Health Qual Life Outcomes. 2007;5. 10.1186/1477-7525-5-63 PMC2222612 18042300

[31] Snell WE. Measuring multiple aspects of the sexual self-concept: the multidimensional sexual self-concept questionnaire. New Dir Psychol Hum Sex Res Theory. 2001;17.

[32] Heidari M, Ghodusi M, Rafiei H (2017). Sexual self-concept and its relationship to Depression, stress and anxiety in Postmenopausal Women. J Menopausal Med.

[33] Ferrer-Urbina R, Sepúlveda-Páez GL, Henríquez DT, Acevedo-Castillo DI, Llewellyn-Alvarado DA (2019). Development and validity evidence of the multidimensional scale of sexual self-concept in a spanish-speaking context. Psicol Reflex e Crit.

[34] Ziaei T, Khoei EM, Salehi M, Farajzadegan Z (2013). Psychometric properties of the Farsi version of modified multidimensional sexual self-concept Questionnaire. Iran J Nurs Midwifery Res.

[35] Ramezani A, Saadat SH, Shams J. Reliability and Validity Assessment of Multi-Dimensional sexual Self- Concept Questionnaire in Iran. December; 2013.

[36] Snell WE, Fisher TD, Walters AS (1993). The multidimensional sexuality questionnaire: an objective self-report measure of psychological tendencies associated with human sexuality. Ann Sex Res.

[37] Rosenberg M (1965). Society and the adolescent self-image.

[38] Ferring D, Filipp S-H (1996). Messung des Selbstwertgefühls: Befunde zu Reliabilität, Validität und Stabilität der Rosenberg-Skala. Diagnostica.

[39] von Collani G, Herzberg PY (2003). Eine revidierte Fassung der deutschsprachigen Skala zum Selbstwertgefühl von Rosenberg. Z für Differ und Diagnostische Psychol.

[40] Tylka TL, Wood-Barcalow NL (2015). The body appreciation scale-2: item refinement and psychometric evaluation. Body Image.

[41] Avalos L, Tylka TL, Wood-Barcalow N (2005). The body appreciation scale: development and psychometric evaluation. Body Image.

[42] Swami V, Stieger S, Haubner T, Voracek M (2008). German translation and psychometric evaluation of the body appreciation scale. Body Image.

[43] Feingold A (2013). A regression framework for effect size assessments in longitudinal modeling of group differences. Rev Gen Psychol.

[44] Fossati C, Torre G, Vasta S, Giombini A, Quaranta F, Papalia R (2021). Physical Exercise and Mental Health: the Routes of a reciprocal relation. Int J Environ Res Public Health.

[45] Mandolesi L, Polverino A, Montuori S, Foti F, Ferraioli G, Sorrentino P (2018). Effects of Physical Exercise on Cognitive Functioning and Wellbeing: Biological and Psychological benefits. Front Psychol.

[46] Penedo FJ, Dahn JR (2005). Exercise and well-being: a review of mental and physical health benefits associated with physical activity. Curr Opin Psychiatry.

[47] Buecker S, Simacek T, Ingwersen B, Terwiel S, Simonsmeier BA (2021). Physical activity and subjective well-being in healthy individuals: a meta-analytic review. Health Psychol Rev.

[48] Weiss LA, Westerhof GJ, Bohlmeijer ET (2016). Can we increase Psychological Well-Being? The Effects of Interventions on Psychological Well-Being: a Meta-analysis of Randomized controlled trials. PLoS ONE.

[49] Hanna JL (2010). Dance and sexuality: many moves. J Sex Res.

[50] Hallam-Jones R, Wylie KR (2001). Traditional dance - A treatment for sexual arousal problems?. Sex Relatsh Ther.

[51] Pai HC, Lee S, Yen WJ (2012). The effect of sexual self-concept on sexual health behavioural intentions: a test of moderating mechanisms in early adolescent girls. J Adv Nurs.

[52] German Psychological Society. Ethische Richtlinien der DGPs und des BDP. Deutsche Gesellschaft für Psychologie e.V.; 2016. https://www.dgps.de/fileadmin/user_upload/PDF/berufsethik-foederation-2016.pdf. Accessed 6 Jun 2023.

[53] European Network of Research Ethics Committees - National Information. : Germany - Short description of RECs system. http://www.eurecnet.org/information/germany.html. Accessed 6 Jun 2023.

