# Supplementary material for: Effects of pole dance on mental wellbeing and the sexual self-concept—a pilot randomized-controlled trial
Source: BMC Psychol. 2023 Sep 14;11:274. doi: 10.1186/s40359-023-01322-z (PMC10503020; doi:10.1186/s40359-023-01322-z)

Effects of Pole Dance on Mental Wellbeing and the Sexual Self-concept - A Pilot Randomized-controlled Trial
Jalda Lena Pfeiffer, Setia Kati Sowitzki, Thomas Schäfer, Frank Euteneuer

Supplement B: Study flow

Figure S1.
Study flow.

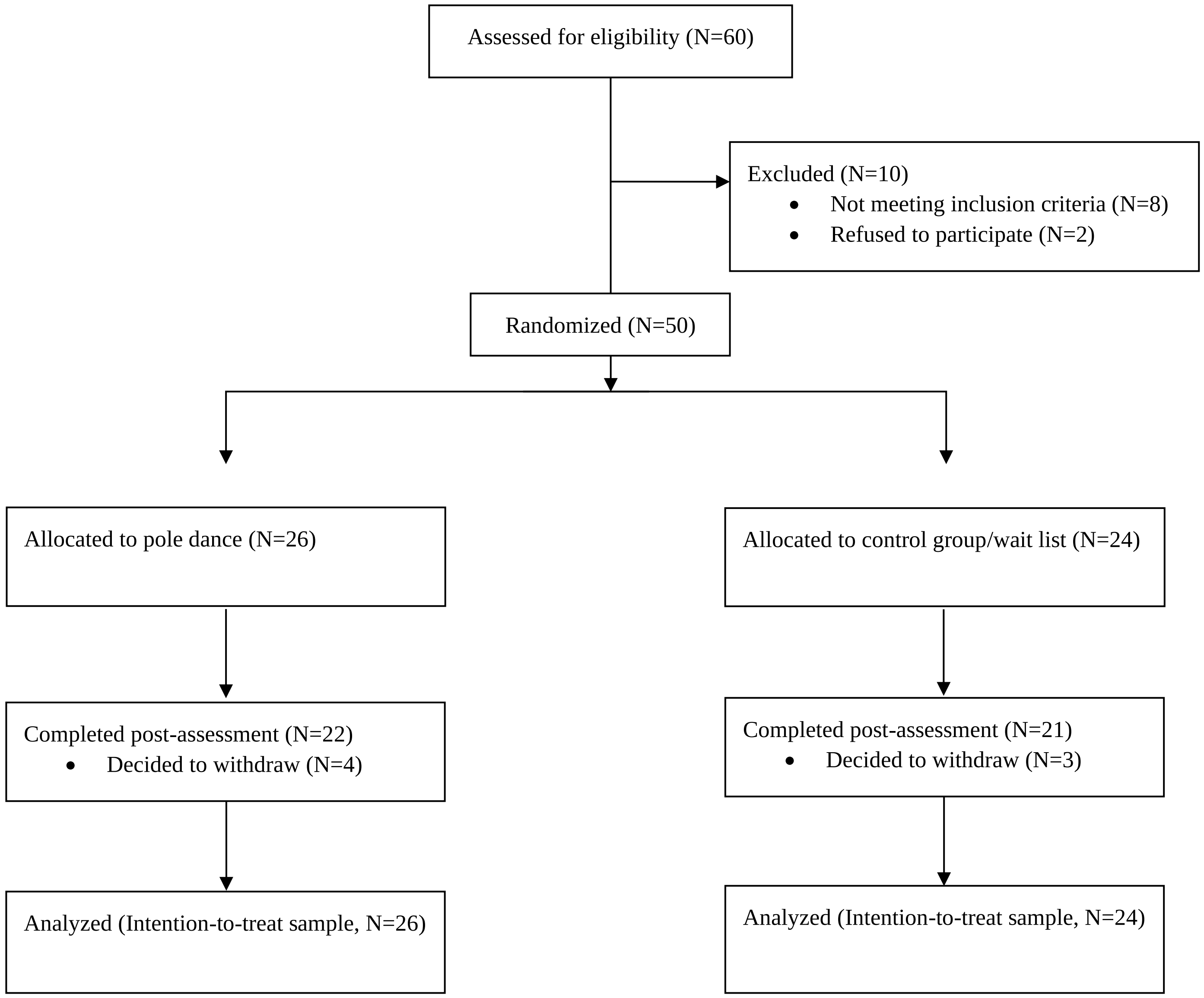

Supplement: Supplementary file 1 — Supplementary Material 1 [file 40359_2023_1322_MOESM1_ESM.pdf]
