# Supplementary material for: Effects of pole dance on mental wellbeing and the sexual self-concept—a pilot randomized-controlled trial
Source: BMC Psychol. 2023 Sep 14;11:274. doi: 10.1186/s40359-023-01322-z (PMC10503020; doi:10.1186/s40359-023-01322-z)
Supplement: Supplementary file 2 — Supplementary Material 2 [file 40359_2023_1322_MOESM2_ESM.pdf]

Effects of Pole Dance on Mental Wellbeing and the Sexual Self-concept - A Pilot Randomized-controlled Trial
Jalda Lena Pfeiffer, Setia Kati Sowitzki, Thomas Schäfer, Frank Euteneuer

Supplement A: Pole dance exercises

Note: The photographs were created by the authors. Exercises are illustrated by author Jalda Lena Pfeiffer. Authors declare that they have not used any copyrighted material, images, or content belonging to others in the creation of these photographs.

The following pole dance specific static and spinning moves (as well as their combinations) were applied:

Session 1

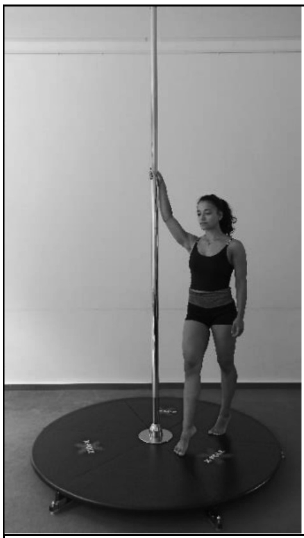 Pole walk	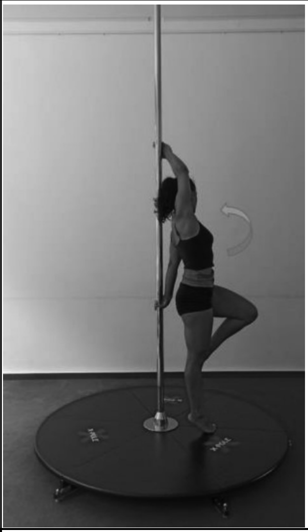 Pirouette
Pole plié 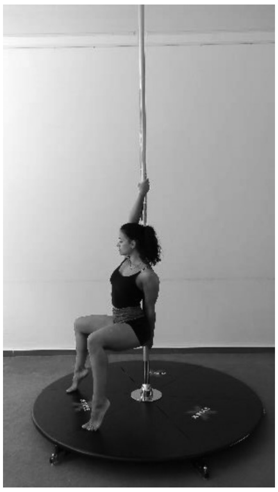 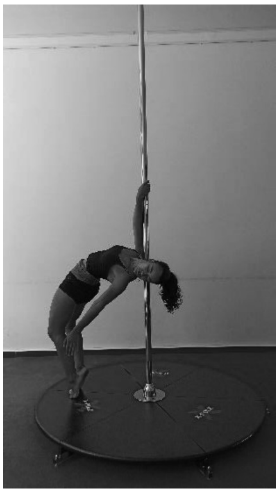	Fireman spin (two variations) 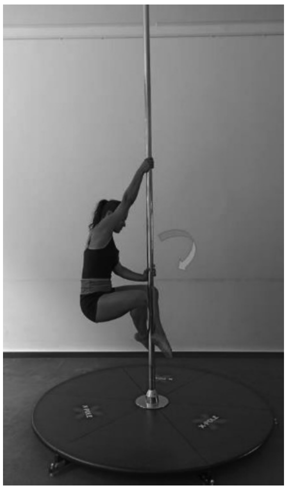 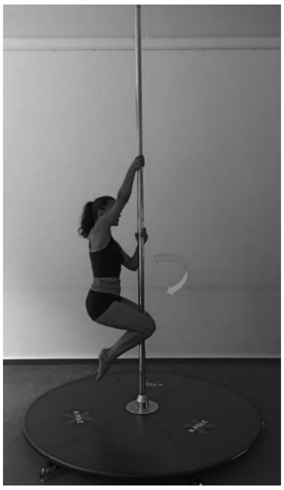

Session 2

Fan kick 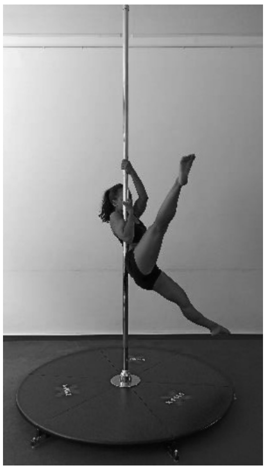 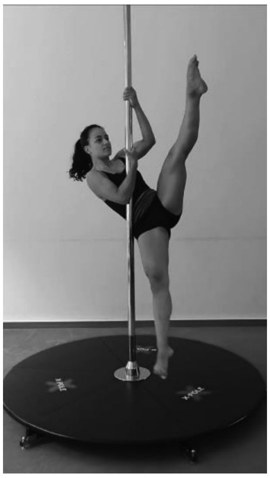	Sit variations (here: mermaid) 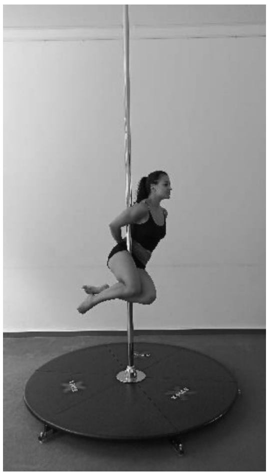
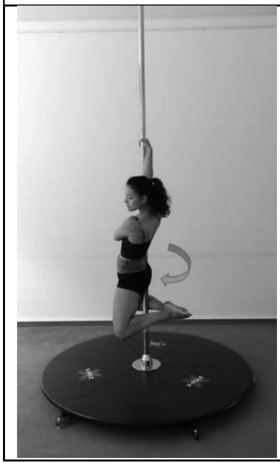 Front hook spin	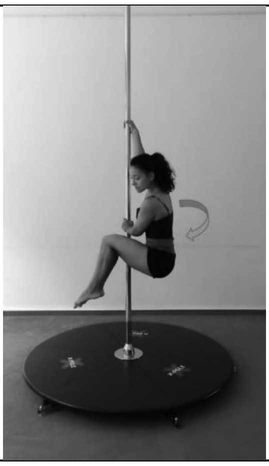 Chair spin

Session 3

Chair spin 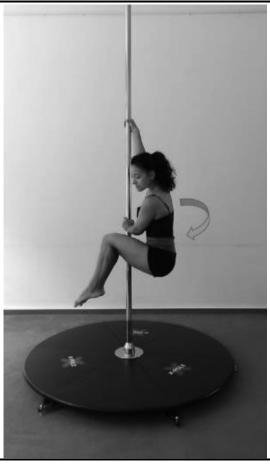	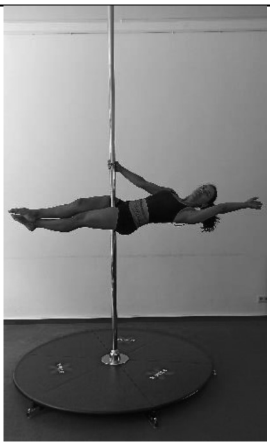 Half plank
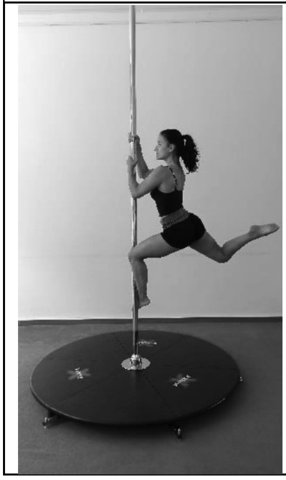 Carousel kick	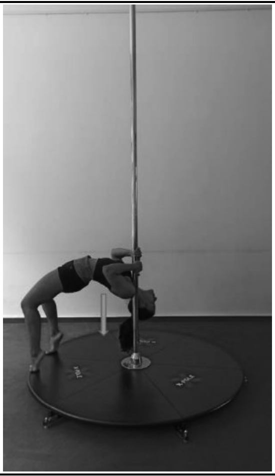 Back slide bridge

Session 4

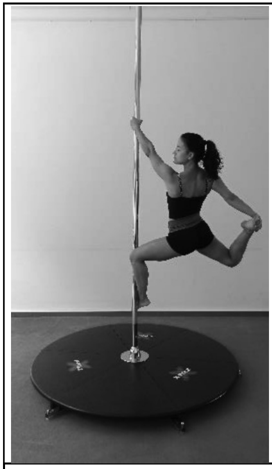 Skater	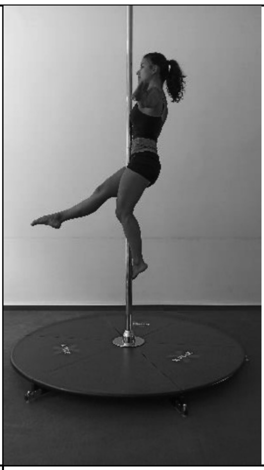 Long sit
Back hook spin 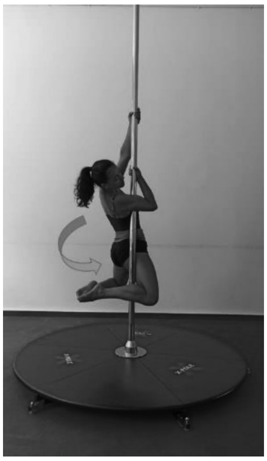	Climb 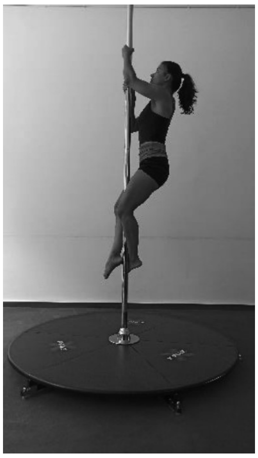 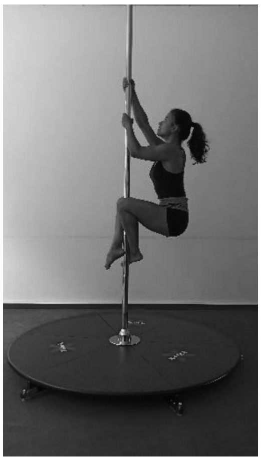

Session 5

Climb 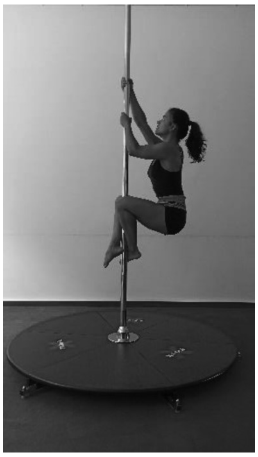 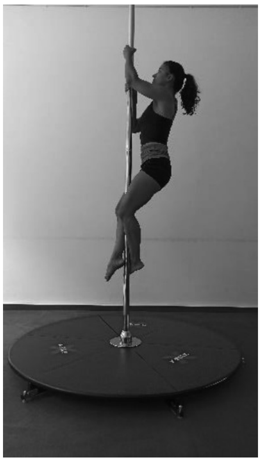	Hero 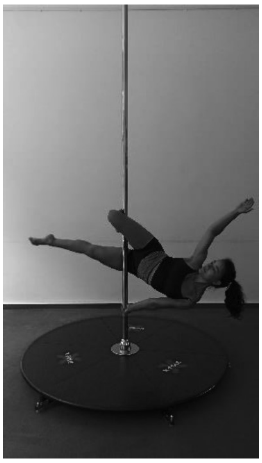
Attitude Spin 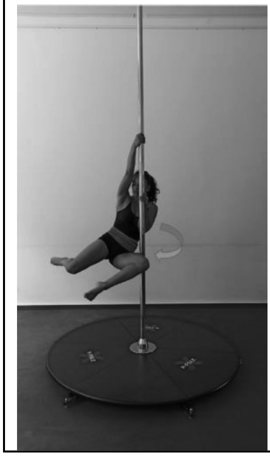	Combination: Front hook spin – Back hook spin

Session 6

Combination: Climb – Carousel kick – Long sit	Head stand (variations) 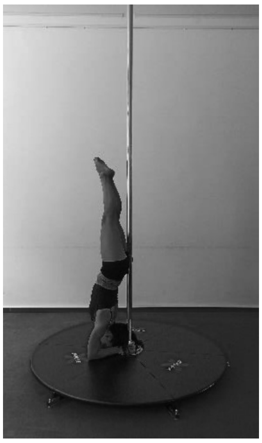 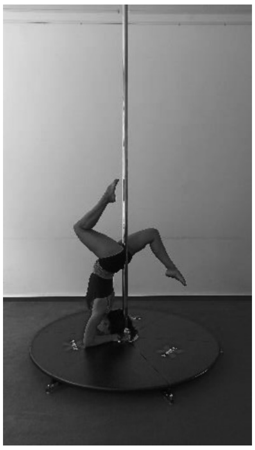
Star gazer 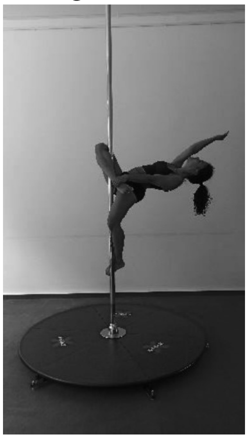	Half plank 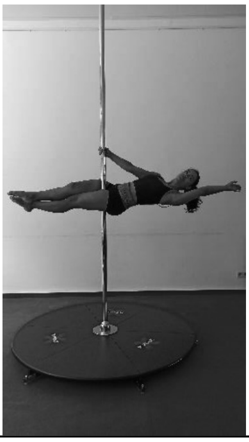

Session 7

Combination: Climb – Star gazer	Crossed knee layback<br>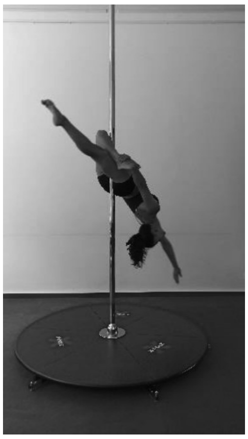
Combination: Hero – Bird<br>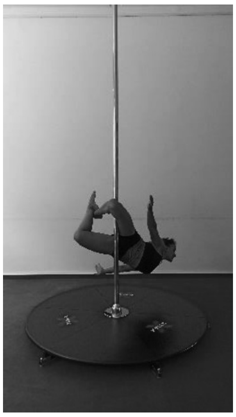	Tinkerbell Spin<br>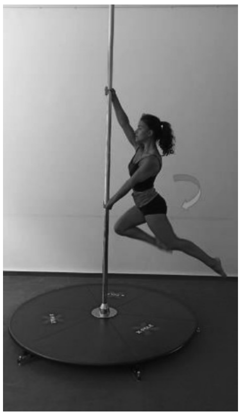

Session 8

Combination: Climb – layback – head stand	Attitude Spin<br>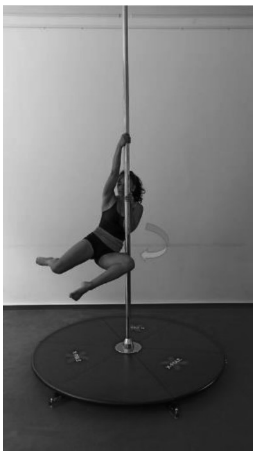
Carousel Spin<br>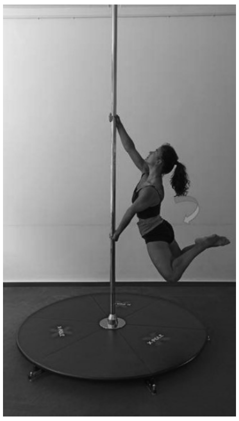	Drama queen<br>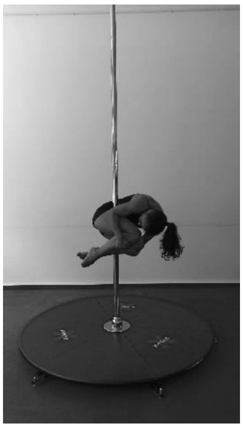
